# Robust Polyurethane with Ordered Hard Segments and Pendant Fluorinated Chains for Improved Hemocompatibility

**DOI:** 10.3390/molecules31111913

**Published:** 2026-06-02

**Authors:** Shengkai Zhao, Rongrong Zhang, Zhaosheng Hou

**Affiliations:** 1 College of Biological Engineering, Qingdao University of Science and Technology, Qingdao 266042, China; sdmuzyp@163.com; 2 College of Chemistry, Chemical Engineering and Materials Science, Shandong Normal University, Jinan 250014, China; 2025214102@stu.sdnu.edu.cn

**Keywords:** polyurethane, fluorinated side chains, ordered hard segments, hemocompatibility

## Abstract

Despite the widespread use of polyurethane (PU) in biomedical devices, its long-term application has been hindered by insufficient hemocompatibility caused by protein adsorption and subsequent thrombosis. In this study, a fluorinated PU (F–PCU) was designed to improve surface hemocompatibility while maintaining mechanical performance and good cytocompatibility. F–PCU was synthesized via a prepolymer method using a fluorinated diol and an ordered hard-segment extender. The chemical structure, thermal behavior, and mechanical properties were systematically characterized, while surface properties and biological performance were evaluated by water contact angle, protein adsorption, platelet adhesion, and cytotoxicity assays. The results demonstrated that fluorinated side chains preferentially enriched at the surface, forming a low-energy interface with significantly enhanced hydrophobicity. F–PCU exhibited excellent mechanical properties with a tensile strength of 49.5 MPa and an elongation at break of 965%. Notably, protein adsorption and platelet adhesion were substantially reduced, while good cytocompatibility was maintained, indicating improved surface hemocompatibility. These findings suggest that integrating ordered hard segments with fluorinated side chains is an effective strategy to optimize both bulk and surface properties, offering promising potential for long-term biomedical applications.

## 1. Introduction

Polyurethane (PU) is a class of multiblock copolymers composed of alternating soft segments (polyether or polyester) and hard segments (diisocyanates) [1]. The polarity difference between soft and hard segments induces microphase separation, in which hard domains are dispersed within the soft matrix and act as physical crosslinking points, thereby endowing PU with excellent mechanical performance, good processability, fatigue resistance, and long-term stability in biological environments [2,3,4,5]. These advantages have led to its widespread application in implantable medical devices, such as ventricular assist devices, vascular grafts, catheters, and pacemaker insulation materials [6,7,8,9]. Despite its generally favorable biocompatibility, PU surfaces tend to induce nonspecific protein adsorption after contact with blood or tissue fluids, which can subsequently trigger platelet adhesion and activation, followed by thrombosis and inflammatory responses. Under long-term implantation conditions, these adverse effects may further cause material degradation, infection, and even device failure, thereby limiting the long-term performance of PU in demanding biomedical applications [10,11]. Therefore, improving the surface hemocompatibility of PU remains a critical objective in the development of biomedical polymers [12,13].

To address these limitations, various modification strategies have been developed, which can be broadly categorized into bulk and surface modification. Bulk modification involves incorporating biocompatible components into the main or side chains of PU through copolymerization, blending, or chemical functionalization, enabling regulation of physicochemical properties and biological behavior at the molecular level [14,15]. This approach offers good uniformity and stability, making the modified material less susceptible to mechanical damage or hydrolytic degradation. However, it often required complex synthesis, and excessive incorporation of functional components may compromise the intrinsic mechanical properties of PU. In contrast, surface modification focuses on the outermost layer of the material, employing techniques such as chemical grafting, plasma treatment, surface coating, and self-assembled monolayers [16,17,18]. This approach enables the introduction of specific biological functions without altering the bulk properties. Nevertheless, the long-term stability of the modified surface remains a major concern, as functional layers may detach, degrade, or be masked by adsorbed biomolecules upon prolonged exposure to physiological conditions. Therefore, achieving a stable and durable improvement in PU biocompatibility remains a significant challenge.

In recent years, fluorinated polymers have attracted considerable attention due to their unique interfacial properties [19,20,21], and the incorporation of fluorinated segments into PU has been regarded as an effective strategy to enhance biocompatibility [22,23,24,25]. Fluorine atoms possess high electronegativity and extremely low surface free energy, which drives their spontaneous migration and enrichment at the material surface during processing. This results in the formation of a fluorine-rich interfacial layer with reduced surface energy, thereby minimizing protein adsorption and suppressing platelet adhesion and activation. In addition, the high bond energy of C–F bonds (~485 kJ/mol) imparts excellent chemical inertness, thermal stability, and resistance to oxidative degradation [26]. Consequently, the fluorinated surface layer is more stable under physiological conditions, which is beneficial for maintaining long-term anti-adhesive performance. Despite these advantages, a key challenge remains in achieving sufficient surface fluorine enrichment while preserving the mechanical integrity of PU under controllable and efficient synthetic conditions.

In this work, a fluorinated PU elastomer with ordered hard segments and pendant fluorinated side chains was designed and synthesized via a conventional prepolymer method. A fluorinated diol chain extender (PEM–diol) was first synthesized through a thiol–ene click reaction between 2-(perfluorooctyl)ethyl methacrylate (PEM) and α-thioglycerol (TGC). Subsequently, PEM–diol and an ordered butanediol–hexamethylene diisocyanate–butanediol (BHB–diol) were employed as co-chain extenders to react with a poly(ε-caprolactone)-based prepolymer, followed by film fabrication via solvent evaporation. PEM–diol introduced surface-active fluorinated side chains, whereas BHB–diol promoted ordered hard-segment formation and mechanical reinforcement. The chemical structures of PEM–diol, BHB–diol, and the resulting fluorinated PU (F–PCU) were systematically characterized. The effects of ordered hard segments on thermal and mechanical properties were investigated by tensile testing, TGA, DSC, and DMA, while the role of fluorinated side chains in surface hemocompatibility was evaluated through protein adsorption and platelet adhesion assays. Furthermore, the cytocompatibility of F–PCU films was evaluated using an in vitro cytotoxicity assay. This molecular design, integrating ordered hard segments with surface-active fluorinated chains, provides an effective strategy for developing high-performance PU materials for long-term biomedical applications.

## 2. Results and Discussion

### 2.1. Characterization of PEM–Diol

**Figure 1:**
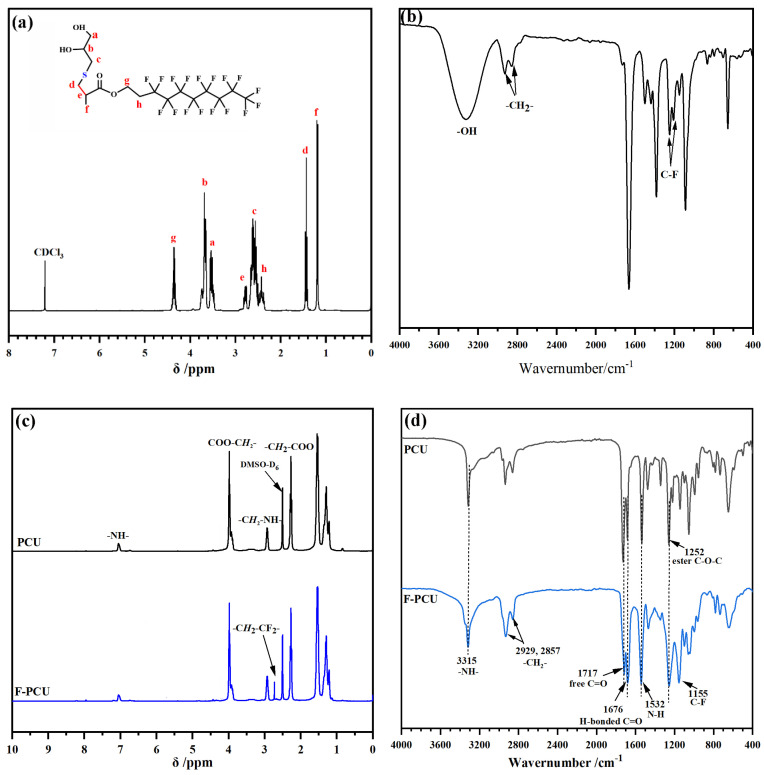
(**a**) NMR and (**b**) FT–IR spectra of PEM–diol; (**c**) NMR and (**d**) FT–IR spectra of PCU and F–PCU films.

Thiol–ene click reactions are widely recognized for their high efficiency, mild reaction conditions, and excellent functional group tolerance, making them highly suitable for the construction of functional molecules [27,28]. Accordingly, a thiol–ene click reaction between the thiol group (–SH) of TGC and alkene group (–C=C) of PEM was employed to introduce terminal diol functionality, yielding PEM–diol. The chemical structure of PEM–diol was confirmed by ^1^H NMR (Figure 1a) and FT–IR (Figure 1b). In the ^1^H NMR spectrum, the characteristic proton signals of –C*H*=C*H*_2_ (δ 6.2 and 5.6 ppm) and –SH (δ 2.1 ppm) disappeared completely, while new signals corresponding to the methylene protons of the thioether linkage (–C*H*_2_–S–C*H*_2_–) appeared at δ 1.43 and 2.61 ppm. The absence of the –OH proton signals was attributed to rapid proton exchange with trace moisture or solvent. These observations confirmed the successful thiol–ene click reaction and the absence of unreacted PEM and TGC. Furthermore, the integral ratios of the characteristic peak were consistent with the expected chemical structure of PEM–diol, verifying its successful synthesis. The FT–IR spectrum further supported this conclusion, showing a characteristic –OH stretching band at 3332 cm^−1^, along with the disappearance of the –SH (~2560 cm^−1^) and –C=C (~1636 cm^−1^) absorption bands. Additionally, a strong doublet at 1250–1206 cm^−1^, assigned to the asymmetric (1250 cm^−1^) and symmetric (1206 cm^−1^) stretching of C–F bonds [29], confirmed the presence of the perfluoroalkyl segment.

### 2.2. Characterization of F–PCU Films

The ^1^H NMR spectra of F–PCU and PCU films are presented in Figure 1c. Both samples exhibited a characteristic resonance at δ 7.1 ppm, corresponding to the N–H protons of the urethane linkages, which was confirmed the successful chain-extension reaction. Signals at δ 4.0 and 2.3 ppm were assigned to methylene protons adjacent to ester groups in the soft segment, corresponding to –COO–C*H*_2_– and –C*H*_2_–COO–, respectively. Compared with PCU, F–PCU displayed an additional signal at δ 2.7 ppm, which was attributed to methylene protons adjacent to the fluorinated alkyl chain (–C*H*_2_–CF_2_–). The appearance of this characteristic resonance indicated the successfully incorporation of fluorinated segments into the polymer chain of F–PCU. Together with the characteristic signals of urethane linkages and soft-segment structure, these results provided convincing evidence for the successful synthesis of fluorinated PCU. To further verify the chemical structure, FT–IR spectroscopy was performed (Figure 1d). Both F–PCU and PCU exhibited characteristic absorption bands at 3315, 1717, 1676, and 1532 cm^−1^, corresponding to the N–H stretching, free C=O stretching, H-bonded C=O stretching, and N–H out-of-plane bending vibration, respectively [30]. These features further confirmed the formation of urethane linkages. In addition, the absorption band at 1252 cm^−1^ was assigned to the C–O–C stretching of the ester groups in the PCL soft segments. It is worth noting that the FT–IR spectrum of F–PCU showed an enhanced and slightly red-shifted band at ~1155 cm^−1^, which was attributed to the H–bonding interactions involving fluorine groups, resulting in a shift of the characteristic vibration to lower wavenumbers. This characteristic feature provided additional evidence for the successful incorporation of fluoroalkyl segments into the polymer chains.

### 2.3. XPS Analysis

**Figure 2:**
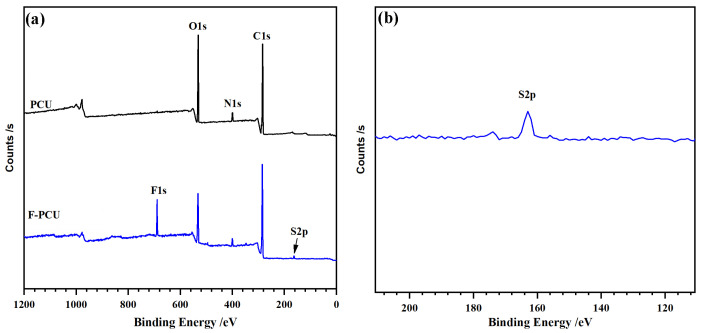
(**a**) Overview and (**b**) narrow-scan XPS spectra of PCU and F–PCU films.

To further verify the successful incorporation of fluorine into the material, the surface elemental composition of the prepared films was analyzed by XPS. The XPS spectra of F–PCU and PCU films are shown in Figure 2a. For the blank PCU film, characteristic peaks located at 532.2, 399.1, and 284.8 eV were observed, corresponding to O 1s, N 1s, and C 1s, respectively. In contrast, the F–PCU films exhibited a significant decrease in the O 1s peak intensity. Meanwhile, new peaks appeared at 689.0 and 163.9 eV, which were assigned to F 1s and S 2p [31], respectively, confirming the successful introduction of fluorinated and sulfur-containing moieties. Although the sulfur content was relatively low, resulting in weak peak intensity, the S2p peak could still be clearly identified in the partially enlarged XPS spectrum of F–PCU (Figure 2b). Notably, the surface fluorine content reached 14.7%, which was significantly higher than the theoretical value of 3.5%. This result indicated that the fluorinated alkyl side chains preferentially migrated and enriched at the film surface during the film-forming process, driven by their strong hydrophobicity. Recent studies on fluorinated acrylate-based coatings have shown that fluoroalkyl side chains can migrate toward the coating–air interface during film formation or curing, leading to fluorine enrichment at the outermost surface and a concomitant reduction in surface energy. For example, Wang et al. reported that fluorinated side chains in crosslinked fluorinated acrylate-modified waterborne PU partially migrated to the coating surface during curing, resulting in surface fluorine enrichment [32]. Similarly, Zhao et al. demonstrated that fluorinated acrylate side-chain structures facilitated fluorine migration to the coating surface, thereby lowering surface energy and improving hydrophobicity [33]. This surface segregation behavior also accounted for the reduced oxygen content at the surface.

### 2.4. Thermal Stability

**Figure 3:**
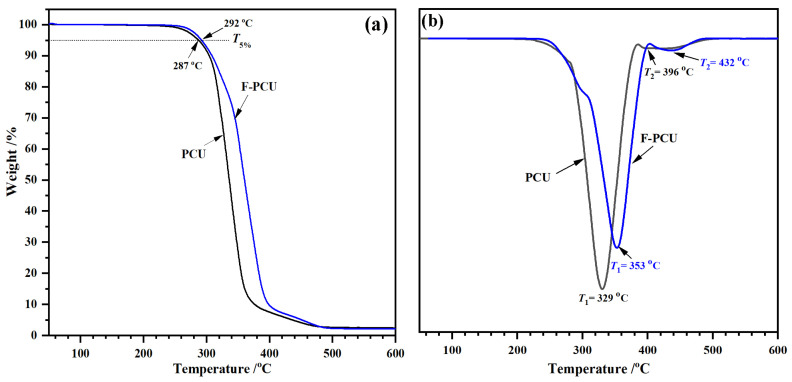
(**a**) TGA and (**b**) DTGA curves of PCU and F–PCU films.

The TGA and DTGA curves of F–PCU and PCU films are presented in Figure 3a and Figure 3b, respectively. As shown in the TGA curves, the temperatures corresponding to 5% weight loss (*T*_5%_) for both F–PCU and PCU were approximately 290 °C, which was much higher than that of PEM–diol (Figure S1), indicating excellent thermal stability. The DTGA curves revealed that both F–PCU and PCU predominantly undergo a single main-stage thermal degradation process. The maximum degradation temperatures (*T*_1_) were observed at 329 °C and 353 °C, respectively, which was attributed to the thermal cleavage of ester bonds in the soft segments and urethane bonds in the hard segments, leading to substantial mass loss. In addition, a minor weight loss was observed at higher temperature (396 °C and 432 °C, denoted as *T*_2_), which could be ascribed to the decomposition of the ether-based initiator used in the synthesis of PCL diol. Compared with PCU, F–PCU exhibited an additional slight weight loss around 300 °C, which was attributed to the cleavage of C–S bonds in the PEM–diol segments due to their relatively low bond dissociation energy. Overall, F–PCU demonstrated slightly improved thermal stability compared to blank PCU. This enhancement can be explained from two aspects [34,35,36]: (1) the strong electronegativity of fluorine atoms induces enhanced interchain dipole–dipole interactions and van der Waals forces, thereby increasing the cohesive energy density of the polymer matrix; (2) the low surface energy of fluorinated groups (e.g., –CF_3_, –CF_2_–) facilitates the formation of a dense fluorocarbon-rich surface layer, which effectively suppresses heat transfer and retards the thermal degradation process.

### 2.5. Thermal Transformation

**Figure 4:**
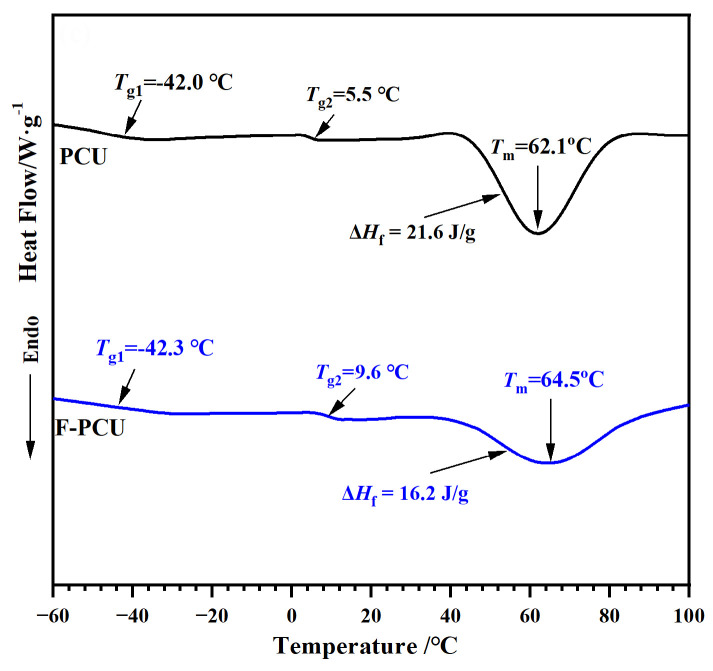
DSC curves of PCU and F–PCU films.

The DSC curves of the F–PCU and PCU films are shown in Figure 4. Both samples exhibited two glass transition temperatures, denoted as *T*_g1_ and *T*_g2_. The *T*_g1_ at a low temperature of approximately −42 °C was associated with the soft PCL segments, whereas the *T*_g2_ appeared at 5.5 and 9.6 °C corresponded to the ordered hard domains [37]. The occurrence of two distinct *T*_g_ values indicated the formation of a microphase-separated morphology, which arose from the thermodynamical immiscibility between the hard and soft segments owing to their intrinsic polarity differences. In addition, a broad endothermic peak was observed at 62~64 °C (*T*_m_), which belonged to the melting of partially crystallized soft and/or ordered hard domains, suggesting the semi-crystalline nature of both films. This interpretation is further supported by the broad and diffuse diffraction peaks observed in the XRD patterns (Figure S2). Compared with blank PCU, F–PCU displayed a broader melting endotherm and a reduced melting enthalpy (Δ*H*_f_ = 16.2 J/g) relative to PCU (Δ*H*_f_ = 21.6 J/g). This decrease in crystallinity can be attributed to the enhanced interchain dipole–dipole interactions induced by fluorine-containing groups, which restrict chain mobility and therefore hinder the formation of well-ordered crystalline domains [38].

### 2.6. Thermomechanical Properties

**Figure 5:**
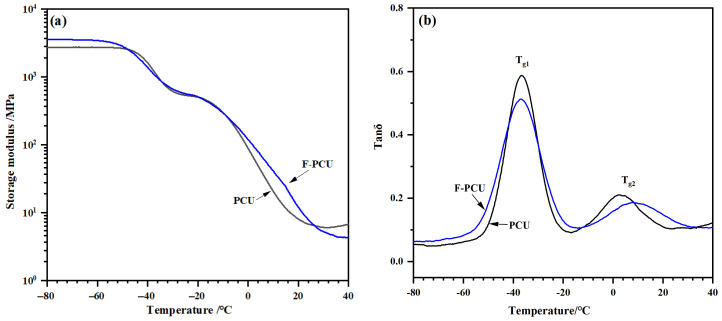
(**a**) Storage modulus (*E*’) and (**b**) tan δ curves of PCU and F–PCU films.

To elucidate the influence of fluorinated groups on the phased-separated and viscoelastic behaviors of the fabricated films, DMA measurements were performed, and the corresponding results are shown in Figure 5. Both PCU and F-PCU films exhibited two distinct relaxation transitions in the tan δ curves (Figure 5b), consistent with the DSC analysis and indicative of a typical microphase-separated structure. The first relaxation peak located at approximately −40 °C was assigned to the glass transition of the soft PCL segments, while the second transition appearing in the range of 0–10 °C corresponded to the relaxation of the ordered hard domains. These observations were in good agreement with the *T*_g_ values obtained from DSC curves, further confirming the thermodynamic immiscibility between the soft and hard segments. In addition, the storage modulus (*E*’) decreased with increasing temperature (Figure 5a), reflecting enhanced segmental mobility during the glass transition process [39]. Compared with PCU, F–PCU showed a relatively broader relaxation behavior and a reduced damping intensity of the soft segment transition, indicating restricted molecular motion caused by fluorine-induced interchain dipole–dipole interactions. Moreover, the slightly lower modulus in the high-temperature region could be attributed to their low crystallinity. The DMA results further demonstrated that the incorporation of fluorinated groups affected the microphase morphology and chain dynamics of the PU matrix.

### 2.7. Mechanical Properties

**Figure 6:**
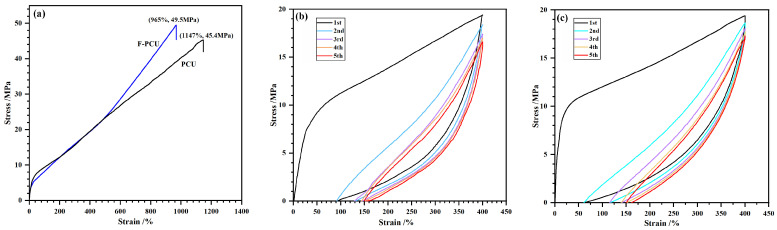
(**a**) Tensile stress–strain curves of PCU and F–PCU films; Cyclic tensile stress–strain curves of (**b**) PCU and (**c**) F–PCU films at 400% strain.

The tensile stress–strain curves of F–PCU and PCU films are shown in Figure 6a. PCU exhibited a maximum tensile strength (ϭ_m_) of 45.4 MPa and an elongation at break (ε_m_) of 1147%, whereas F–PCU showed corresponding values of 49.5 MPa and 965%, indicating that both materials possessed excellent mechanical properties. This behavior was primarily attributed to the presence of well-ordered hard segments within the PU matrix. The urethane-rich hard segments form abundant H-bonds between C=O and N–H groups, generating a physically crosslinked network that facilitates efficient stress transfer [40]. Upon stretching, intermolecular H-bonds undergo reversible dissociation and reformation, enabling energy dissipation and chain rearrangement, thereby enhancing both the strength and stretchability. Compared with PCU, F–PCU exhibited a slightly higher ϭ_m_ but a reduced ε_m_. This difference can be ascribed to the incorporation of fluorinated side chains, which strengthen intermolecular interactions (e.g., weak H–bonding or dipolar interactions), leading to a more compact microstructure and reduced chain mobility.

To further evaluate the mechanical behaviors, cyclic tensile tests were conducted (Figure 6b,c). Both materials exhibited pronounced hysteresis during cyclic loading–unloading, which was attributed to internal friction and irreversible chain rearrangement. During stretching, polymer chains must overcome intermolecular interactions to reorient, resulting in energy dissipation; upon unloading, incomplete recovery of chain configurations leads to a distinct hysteresis loop. Similar behavior had been reported for segmented PU with ordered hard segments [41]. Under constant strain, the maximum stress (ϭ_c-m_) decreased significantly in the initial cycles and gradually stabilized with further cycling. After five cycles, F–PCU retained 89.1% of its initial stress, which was higher than that of PCU (84.8%), indicating relatively superior fatigue resistance. This improvement is attributed to the introduction of fluorine, which enhances intermolecular interactions and restricts chain slippage, thereby promoting a elastic recovery during cyclic deformation.

### 2.8. Surface Hydrophobicity

**Figure 7:**
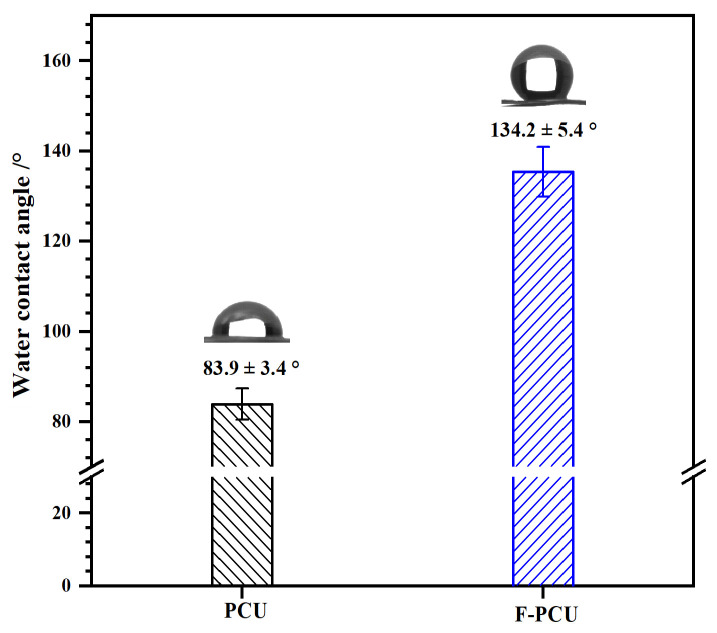
Water contact angles on PCU and F–PCU film surface.

Surface wettability is closely associated with hemocompatibility, as either increased hydrophilicity or hydrophobicity can effectively reduce protein and platelet adhesion. WCA measurement is commonly used to characterize surface wettability. As shown in Figure 7, the blank PCU exhibited a WCA of 83.9 ± 3.4°, indicating moderate surface hydrophobicity. Upon introduction of fluorinated alkyl side chains, the WCA of F–PCU increased markedly to 134.2 ± 5.4°, demonstrating significantly enhanced surface hydrophobicity. This enhancement can be attributed to the unique physicochemical properties of fluorinated alkyl groups. On the one hand, fluorinated chains possess extremely low surface energy and tend to migrate and enrich at the surface during film formation, therefore forming a dense hydrophobic layer. Compared with conventional fluorinated PU systems [42], in which fluorinated segments are typically incorporated into the backbone, the present design featuring flexible pendant fluorinated side chains provides higher chain mobility, thus facilitating surface segregation of the fluorinated side moieties [43]. On the other hand, the high bond energy and low polarizability of C–F bonds weaken H–bonding and van der Waals interactions with water molecules, thereby inhibiting water spreading and increasing contact angle. However, the WCA of F–PCU has not yet reached the superhydrophobic regime (typically ≥ 150°). Therefore, further optimization of hydrophobic modification is required while maintaining mechanical performance, such as by optimizing the compositional ratio of BHB–diol and PEM–diol or introducing longer fluorinated alkyl chains, which will be explored in future work.

### 2.9. Surface Hemocompatibility

When blood contacts a foreign surface, thrombus formation is initiated by the adsorption of plasma proteins, followed by platelet adhesion and activation [44,45]. Therefore, protein adsorption and platelet adhesion are commonly regarded as primary and straightforward approaches to evaluate surface hemocompatibility.

#### 2.9.1. Protein Adsorption

**Figure 8:**
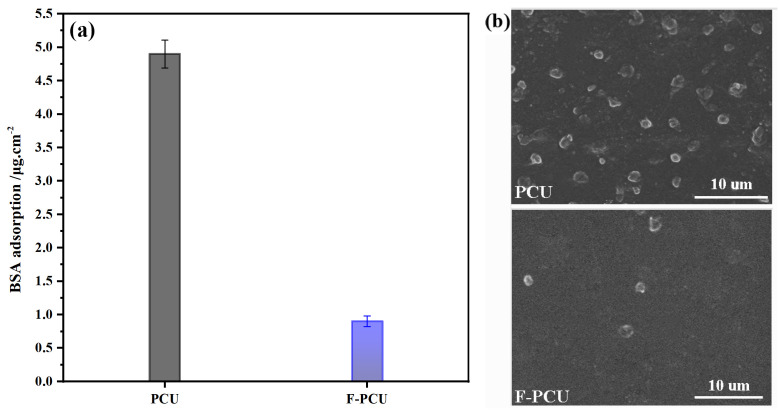
(**a**) Protein adsorption and (**b**) platelet adhesion on PCU and F–PCU film surface.

The extent of BSA adsorption on the F–PCU and blank PCU films is presented in Figure 8a. The F–PCU surface exhibited a markedly reduced BSA amount of 0.9 ± 0.08 μg/cm^2^, in contrast to the significantly higher value measured for the PCU surface (4.9 ± 0.21 μg/cm^2^). Surfaces with low interfacial free energy are known to effectively suppress the adsorption of plasma proteins [46]. In this work, the fluorinated modification endowed the surface with pronounced hydrophobicity and consequently lowered its interfacial energy, thereby minimizing protein–surface interactions and leading to a substantial decrease in protein adsorption. Notably, the adsorption level on F–PCU was significantly lower than that reported for silicone-coated PU (~2.9 μg/cm^2^) [47]. Although further protein adsorption studies involving physiologically relevant proteins or media (such as fibrinogen or diluted plasma) are required to more comprehensively assess blood-contacting performance, the markedly reduced BSA adsorption suggests that the modified F–PCU surface has promising hemocompatibility.

#### 2.9.2. Platelet Adhesion

The morphology of platelets adhered to the F–PCU and PCU film surfaces was characterized by SEM, and representative images are presented in Figure 8b. The blank PCU surface exhibited extensive platelet adhesion, with a quantified density of approximately 39,800 platelets/mm^2^. In addition, many of the adhered platelets displayed pronounced morphological changes, including spreading and pseudopodia extension, suggesting platelet activation. In contrast, the F–PCU surface demonstrated a markedly reduced level of platelet adhesion, with the platelet density decreasing to 4729 platelets/mm^2^ based on statistical analysis. Moreover, most adhered platelets retained their native discoid morphology, with no evident deformation or aggregation, suggesting that platelet activation was effectively inhibited. A similar phenomenon has also been reported for PDMS-coated PU surfaces [48]. These findings demonstrated that the fluorinated surface effectively inhibited platelet adhesion and activation. In blood-contacting environments, polymer surfaces are rapidly covered by adsorbed proteins, and subsequent platelet adhesion and activation are largely governed by the amount, composition, and conformation of the adsorbed protein layer [49,50]. Therefore, the improved anti-platelet adhesion behavior of F–PCU can be attributed to the fluorinated low-surface-energy interface, which reduces nonspecific protein adsorption and may further modulate the interfacial protein layer, thereby suppressing platelet adhesion and activation.

### 2.10. Cytotoxicity

**Figure 9:**
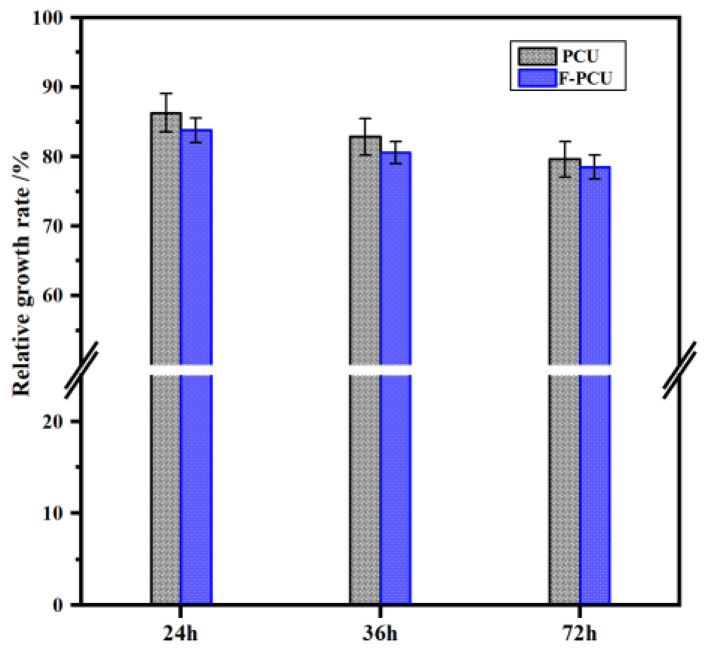
Relative growth rates of L929 cells in PCU and F–PCU extracts.

Cytotoxicity assessment is an essential in vitro evaluation for determining the biological safety of biomaterials prior to further biomedical application. In this study, the cytocompatibility of the prepared films was investigated by culturing L929 fibroblasts with the corresponding material extracts, followed by an MTT assay. The relative growth rates (RGRs) after incubation for predetermined periods are displayed in Figure 9. Compared with the PCU sample, F–PCU showed slightly lower RGR values at all tested time points, which should be associated with the compositional modification of the surface after fluorination. Nevertheless, the RGR values of cells exposed to F–PCU extracts remained above 75% throughout the incubation period, indicating that the F–PCU film did not markedly inhibit cell viability or proliferation. According to the cytotoxicity grading criteria defined in ISO 10993–5:2016 [51], materials with RGR values higher than 75% are classified as grade 1, corresponding to a slight cytotoxic response. Therefore, F–PCU exhibited favorable cytocompatibility and acceptable biological safety, supporting its potential application as an implantable biomaterial.

## 3. Materials and Methods

### 3.1. Materials

Poly(ε-caprolactone) diol (PCL–diol, M_n_ = 2000 g/mol) was purchased from J&K Scientific Ltd. (Beijing, China) and was dehydrated at 90 °C under vacuum for 3 h. Stannous octoate ((Oct)_2_Sn, >95%), N,N-diisopropylethylamine (DIPA, >99.5%), PEM (>98%), and TGC (>99%) were obtained from Sigma-Aldrich (St. Louis, MO, USA). 1,4-Butanediol (BDO, >99%), hexamethylene diisocyanate (HDI, 99%), and anhydrous N,N-dimethylformamide (DMF, moisture content < 0.05%) were supplied by Aladdin Reagent Co. (Shanghai, China). Fresh anticoagulated rabbit blood was obtained from a commercial supplier (Success Bio-Tech Co., Ltd., Jinan, China). Other reagents were of analytical grade and purified by standard methods.

### 3.2. Synthesis of BHB–Diol

BHB–diol was synthesized according to our previously reported method [52]. Briefly, HDI was treated with an eight-fold molar excess of BDO at 80 °C for 3 h in the absence of a catalyst. After cooling to room temperature, the reaction mixture was washed three times with dry acetone to remove excess BDO. The product was then dried under reduced pressure to constant weight, giving a white powder with a yield of 93.5%. The chemical structure of BHB–diol was confirmed by ^1^H NMR (Figure S3), ^13^C NMR (Figure S4), and HR–MS (Figure S5).

### 3.3. Synthesis of PEM–Diol

**Figure 10:**
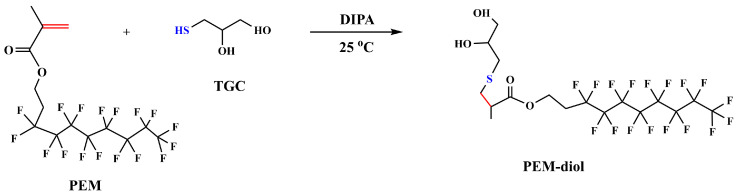
Synthesis of PEM–diol by thiol–ene click reaction.

PEM–diol was synthesized via a thiol–ene click reaction between PEM and TGC (Figure 10). Specifically, PEM (10.64 g, 20 mmol) and TGC (2.38 g, 22 mmol) were dissolved in tetrahydrofuran (THF, 25 mL), followed by the addition of DIPA (0.26 g, 0.2 mmol) as a catalyst. The reaction was carried out at room temperature in the dark for 24 h. After completion, the reaction mixture was poured into cold diethyl ether (250 mL, 0 °C) to precipitate the product. The obtained product was collected and dried under reduced pressure at room temperature to give PEM–diol as a pale-yellow viscous liquid with a yield of 94.7%.

### 3.4. Preparation of F–PCU and F–PCU Film

**Figure 11:**
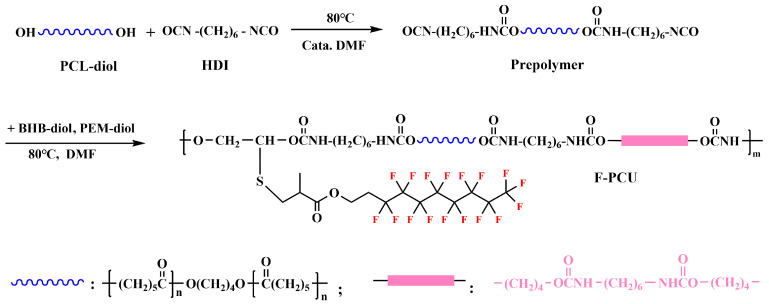
Synthetic route of F–PCU.

F–PCU was synthesized via a prepolymer chain extension approach, as illustrated in Figure 11. PCL–diol (20 g, 10 mmol) and HDI (3.53 g, 21 mmol) were dissolved in anhydrous DMF (20 mL) under mechanical stirring. After the addition of (Oct)_2_Sn (0.2 wt% relative to PCL–diol) under a dry argon atmosphere, the reaction was conducted at 80 °C until the –NCO content, determined by dibutylamine titration, reached the theoretical value (approximately 2.5 h). Subsequently, a DMF solution (16.8 mL) containing BHB–diol (2.6 g, 7.5 mmol) and PEM–diol (1.6 g, 2.5 mmol) was added dropwise under vigorous stirring. The reaction was continued at 80 °C until the complete disappearance of the -NCO absorption peak in the FT–IR spectrum. The resulting solution was diluted with DMF to a solid content of 5 wt%, degassed under reduced pressure, and gently cast into a Teflon mold. The solvent was evaporated at 45 °C for 48 h, followed by vacuum drying to remove residual solvent, yielding semitransparent F–PCU films with a thickness of 0.2 ± 0.02 mm (Figure S6). For comparison, a fluorine-free PU (named as PCU) was prepared following the identical procedure, except that BHB–diol was used as the sole chain extender. GPC for F–PCU: $\bar{M}$_w_ = 88,300 g/mol, $\bar{M}$_n_ = 67,100 g/mol, *Đ*_M_ = 1.32; for PCU: $\bar{M}$_w_ = 92,500 g/mol, $\bar{M}$_n_ = 75,600 g/mol, *Đ*_M_ = 1.22.

### 3.5. Instruments and Characterization

^1^H/^13^C NMR spectra were recorded on a Bruker Avance II 400 MHz spectrometer (Rheinstetten, Germany) using CDCl_3_ or DMSO-d_6_ as the solvent. FT–IR spectra were obtained on an Alpha ATR-FTIR spectrometer (Rheinstetten, Germany) over the range of 4000–400 cm^−1^. Molar mass ($\bar{M}$_w_, $\bar{M}$_n_) and dispersity (*Đ*_M_) were determined by GPC (Waters Alliance GPC2000, Milford, MA, USA) with THF as the eluent and monodispersed polystyrene standards for calibration.

Surface elemental composition was analyzed using an ES CALAB 250 XPS (Thermo Scientific, Waltham, MA, USA) at a take-off angle of 90°. A monochromatic Al-Kα radiation source (1466.8 eV) was employed.

Thermal stability was evaluated by TGA (Q50, TA Instruments, New Castle, DE, USA) under nitrogen from 40 to 600 °C at a heating rate of 10 °C/min. DSC (DSC25, TA Instruments, New Castle, DE, USA) was used to investigate thermal transitions. Samples were first heated to erase thermal history and then subjected to a second heating scan from –60 to 100 °C at 10 °C/min under nitrogen. Dynamic mechanical analysis (DMA) was performed using a Q800 dynamic mechanical analyzer (TA Instruments, New Castle, DE, USA) in tensile mode. The tests were conducted at a frequency of 1 Hz with a heating rate of 3 °C/min over a temperature range of −80 to 40 °C.

Water contact angle (WCA) was measured using a CAM200 contact angle goniometer (KSV, Helsinki, Finland). Prior to testing, the surface of the sample was washed with ethanol and dried. A 2 μL water droplet was deposited onto the surface, and images were captured within 3 s.

Mechanical properties were measured according to GB/T 1040.3–2006 [53]. Samples were cut into a dumbbell shape (width: 4.0 mm; gauge length: 30 mm) and tested at a crosshead speed of 20 mm/min. Cyclic tensile tests were conducted up to 400% strain for five loading–unloading cycles.

Protein adsorption on the film surface was quantified using a Bradford assay, with bovine serum albumin (BSA) as a model protein, following previously reported methods [54,55]. Circular samples (~10 mm in diameter) were incubated in 1.0 mL BSA solution (45 μg/mL) at 37 °C for 120 min. After incubation, the samples were removed and rinsed with phosphate-buffered saline (PBS) to eliminate loosely bound proteins. The adsorbed proteins were then desorbed by ultrasonication in 1 wt% sodium dodecyl sulfate solution at 37 °C for 30 min. The BSA concentration was determined using a micro-Bradford assay kit, and absorbance was measured at 595 nm on a microplate reader. The amount of adsorbed protein was calculated based on a standard calibration curve.

Platelet-rich plasma (PRP) was prepared from freshly collected rabbit blood anticoagulated with sodium citrate. Film disks (~10 mm in diameter) were preconditioned in PBS (pH 7.4) for 12 h and then incubated with 1.0 mL PRP at 37 °C for 60 min. After incubation, nonadherent platelets were removed by gentle rinsing with PBS. The attached platelets were fixed with 2.5% glutaraldehyde at 37 °C for 30 min, followed by washing with PBS and dehydration through a graded ethanol series (50–100%, *v*/*v*). The samples were air-dried, sputter-coated with gold, and observed by SEM (SU–8010, Hitachi, Tokyo, Japan). Adherent platelets were quantitatively analyzed by calculating surface coverage from SEM images. At least eight random areas were selected for each sample.

In vitro cytotoxicity was evaluated using the MTT assay with L929 mouse fibroblasts as test cells, in accordance with ISO 10993-5:2016 [51].

## 4. Conclusions

In this study, a fluorinated polyurethane elastomer (F–PCU) with ordered hard segments and pendant fluorinated side chains was successfully synthesized via a prepolymer method. XPS characterization revealed significant fluorine enrichment at the surface, resulting in the formation of a low-energy interface. Compared with fluorine-free PCU, F–PCU maintained excellent thermal stability and mechanical performance. Moreover, the incorporation of fluorinated side chains markedly enhanced surface hydrophobicity and reduced protein adsorption and platelet adhesion, indicating improved hemocompatibility. In addition, F–PCU exhibited good cytocompatibility, further supporting its potential suitability for biomedical applications. Although further biological evaluations are still required to comprehensively assess its long-term biocompatibility and in vivo performance, these findings demonstrate that the integration of ordered hard segments with fluorinated side chains provides an effective molecular design strategy for simultaneously achieving robust mechanical properties, improved hemocompatibility, and favorable cytocompatibility in PU materials for potential long-term biomedical applications.

## Data Availability

All data are contained within the manuscript and are available upon request.

## Supplementary Materials

The following supporting information can be downloaded at: https://www.mdpi.com/article/10.3390/molecules31111913/s1. Figure S1. TGA curve of PEM-diol (heating rate: 5 °C/min). Figure S2. XRD patterns of PCU and F-PCU films. Figure S3. ^1^H NMR spectrum of BHB-diol. Figure S4. ^13^C NMR spectrum of BHB-diol. Figure S5. HR-MS spectrum of BHB-diol. Figure S6. Image of F-PCU film.
