# Supplementary material for: Robust Polyurethane with Ordered Hard Segments and Pendant Fluorinated Chains for Improved Hemocompatibility
Source: Molecules. 2026 Jun 2;31(11):1913. doi: 10.3390/molecules31111913 (PMC13258626; doi:10.3390/molecules31111913)
Supplement: Supplementary file 1 [file molecules-31-01913-s001.zip › molecules-4295337-supplementary.pdf]

Supplementary Materials

Robust Polyurethane with Ordered Hard Segments and Pendant Fluorinated chains for Improved Hemocompatibility

Shengkai Zhao[1], Rongrong Zhang[2], and Zhaosheng Hou [2,*]

[1.] College of Biological Engineering, Qingdao University of Science and Technology, Qingdao 266042, PR China; sdmuzyp@163.com (S. Z)

[2.] College of Chemistry, Chemical Engineering and Materials Science, Shandong Normal University, Jinan 250014, PR China; 2025214102@stu.sdnu.edu.cn (R. Z.); houzs@sdnu.edu.cn (Z.H.)

* Correspondence: houzs@sdnu.edu.cn (Z.H.)

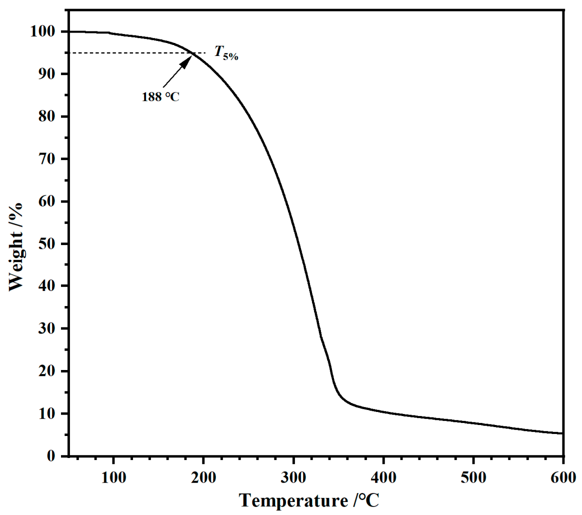

Figure S1. TGA curve of PEM-diol (heating rate: 5 °C/min).

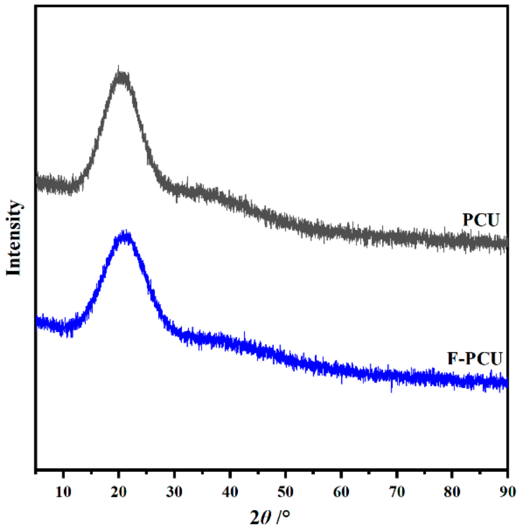

Figure S2. XRD patterns of PCU and F-PCU films.

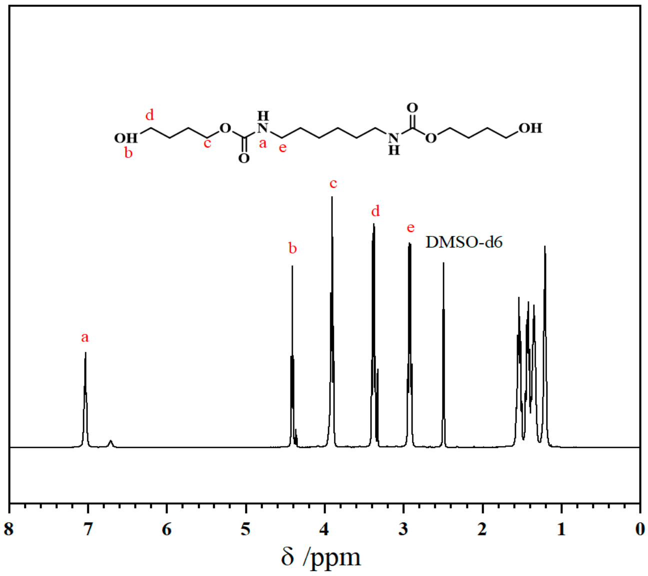

Figure S3. ^{1}H NMR spectrum of BHB-diol.

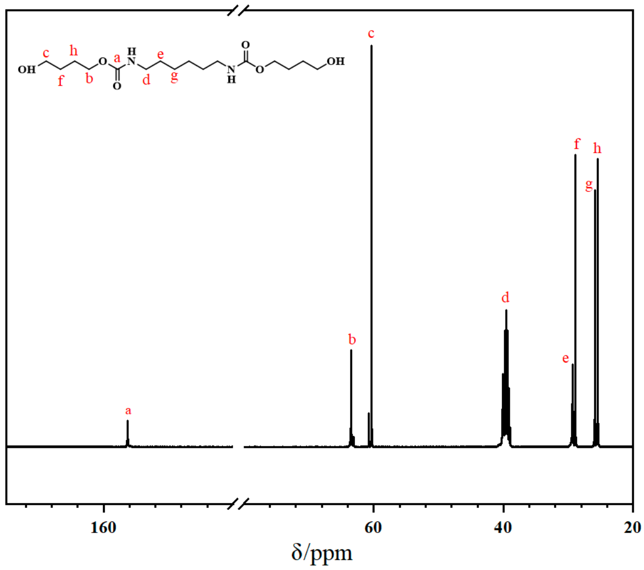

Figure S4. ^{13}C NMR spectrum of BHB-diol.

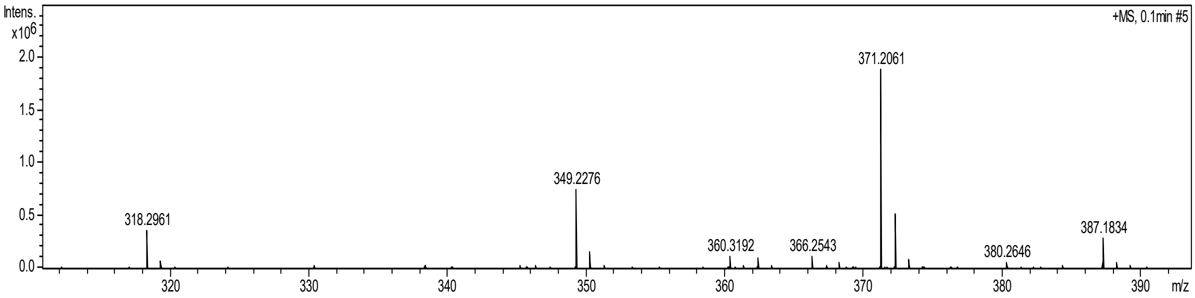

Figure S5. HR-MS spectrum of BHB-diol.

(Calculated for $C_{16}H_{32}O_6N_2$ [M + Na^{+}] 371.4256, found 371.2061)

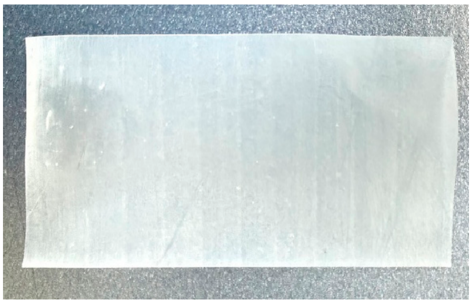

Figure S6. Image of F-PCU film.
